# Genome Annotations of Two *Bacillus* Phages, Tomato and BaseballField

**DOI:** 10.1128/MRA.01196-20

**Published:** 2021-01-07

**Authors:** Julie Duperier, Maikah Bulpitt, Felipe Bispo, Elizabeth Greguske

**Affiliations:** a Department of Biology, Saint Anselm College, Manchester, New Hampshire, USA; Queens College

## Abstract

Tomato and BaseballField are *Bacillus* bacteriophages that were isolated and annotated by students in the Howard Hughes Medical Institute Phage Hunters program. Tomato has a unique truncation of the tape measure gene that is not found in other closely related C1 *Bacillus* phages. BaseballField is a strictly lytic phage with a compact genome of 26 kb.

## Abstract (precis)

Tomato and Baseball Field are *Bacillus* bacteriophages that were isolated and annotated by students in the Howard Hughes Medical Institute Phage Hunters program. Tomato has a unique truncation of the tape measure gene that is not found in other closely related C1 *Bacillus* phages. Baseball Field is a strictly lytic phage with a compact genome of 26 kb.

## ANNOUNCEMENT

*Bacillus* phages Tomato and BaseballField were isolated, sequenced, and annotated by students in the Howard Hughes Medical Institute (HHMI) Phage Hunters program (1). Tomato was isolated from the soil of a commercially purchased tomato plant. BaseballField was isolated from the soil in the outer left field of the varsity baseball field of Saint Anselm College (Manchester, NH, USA). Five grams of soil was put into a 45-ml log-phase culture of Bacillus thuringiensis serovar kurstaki in tryptic soy broth (TSB) and incubated overnight at 37°C, with shaking. Cultures were centrifuged at 3,000 rpm for 10 min, the supernatant was decanted, and 3-ml samples were sterile filtered through a 0.22-µm-pore syringe filter (2). Individual phages were isolated via spot test on tryptic soy agar (TSA) and passaged three times to ensure purity (2). For DNA isolation, phages were grown overnight at 37°C, with shaking, in 10 ml of log-phase B. thuringiensis serovar kurstaki and sterile filtered as described above. A high titer (>3 × 10^8^ PFU/ml) was confirmed via serial dilution in SM buffer (2) and subsequent plate counting. DNA was isolated from purified high-titer phage using the Promega DNA Wizard kit and sequenced via the HiSeq 2500 Illumina platform at the Hubbard Center for Genome Studies (University of New Hampshire, Durham, NH, USA). Tomato contained 2,580,416 paired-end reads. BaseballField contained 1,381,506 paired-end reads. Both phages had an average read length of 251 bp. The reads were trimmed by Trimmomatic v0.33 with default settings (3). *De novo* assembly was completed for both genomes using Geneious v2020.1 (4) with default settings except for sensitivity, which was set to highest sensitivity/slow. The average depth of coverage for Tomato was 1,360.2×, and that for BaseballField was 735.93×. Open reading frames were identified by Glimmer3, used as a plugin in Geneious with default settings.

Tomato was autoannotated by Geneious v2020.1 using *Bacillus* phage Saddex (GenBank accession number MH538193) (5), followed by a manual cross-check with five other *Bacillus* phage genomes available in GenBank (6, 7). The genome of Tomato is 146,370 bp long with a G+C content of 38.9%. Of the 200 predicted proteins, 58 had an identified function, almost all of which had analogs in other *Bacillus* phages (5–7). A unique truncation of the tape measure gene was found in Tomato but was not found in other closely related *Bacillus* phages, indicating that this gene might be an area of further interest. Five tRNAs were identified, all of which had 99 to 100% similarity to tRNAs found in other *Bacillus* phages (5–7).

BaseballField was autoannotated by Geneious v2020.2 using three *Bacillus* phages, namely, SerPounce (GenBank accession number KY947509.1) (8), followed by a visual cross-check with StevenHerd11 (GenBank accession number MK084630.1) and Claudi (GenBank accession number KX349900.1). BaseballField is 26,863 bp with a G+C content of 30.4%. A total of 32 predicted genes were identified, of which 15 had assigned functions, typically for tail or capsid structures. No endonucleases, or any tRNAs, were identified, suggesting that BaseballField is a strictly lytic phage and lacks the ability to undergo lysogeny.

The *Bacillus* phages are clustered using average nucleotide identity (ANI) as proposed by Grose et al. (9). Tomato falls into the C1 cluster, with a mean ANI of 92% when compared to six other C1 phages using BLASTn (10). BaseballField shares 94% ANI with two other unclustered phages, StevenHerd11 and Claudi, suggesting that a new cluster may be emerging.

### Data availability.

The complete genome sequences of the *Bacillus* phages Tomato and BaseballField are available in GenBank with accession numbers MT584805 and MT777452, respectively. Raw reads are available in the SRA; the accession number for Tomato is PRJNA603011, and that for BaseballField is PRJNA646942. Tomato and BaseballField are also registered with the HHMI Phage Hunters program (SEA-PHAGES) (bacillus.phagesdb.org) (11).
